# Palliative Care in Nursing Home Residents with Young-Onset Dementia: Professional and Family Caregiver Perspectives

**DOI:** 10.3233/JAD-230486

**Published:** 2024-01-16

**Authors:** Jasper Maters, Jenny T. van der Steen, Marjolein E. de Vugt, Christian Bakker, Raymond T.C.M. Koopmans

**Affiliations:** a Department of Primary and Community Care, Radboud University Medical Center, Nijmegen, the Netherlands; b Radboudumc Alzheimer Center, Nijmegen, the Netherlands; c Department of Public Health and Primary Care, Leiden University Medical Center, Leiden, the Netherlands; d School for Mental Health and Neuroscience, Alzheimer Center Limburg, Maastricht University Medical Center, Maastricht, the Netherlands; e Groenhuysen, Center for Geriatric Care, Roosendaal, the Netherlands; f Joachim en Anna, Center for Specialized Geriatric Care, Nijmegen, the Netherlands

**Keywords:** Advance care planning, Alzheimer’s disease, palliative care, young-onset dementia

## Abstract

**Background:**

The evidence underpinning palliative care in dementia is mostly based on research in older populations. Little is known about the palliative care needs of people with young-onset dementia (YOD).

**Objective:**

To describe palliative care practices including advance care planning (ACP) in people with YOD residing in Dutch nursing homes.

**Methods:**

The study presents baseline questionnaire data from an observational cohort study. Physicians, family caregivers, and nursing staff completed questionnaires about 185 residents with YOD. The questionnaires included items on sociodemographics, quality of life measured with the quality of life in late-stage dementia (QUALID) scale, dementia-related somatic health problems, symptoms, pain medication, psychotropic drugs, and ACP.

**Results:**

The mean age was 63.9 (SD 5.8) years. Half (50.3%) of them were female. Alzheimer’s disease dementia (42.2%) was the most prevalent subtype. The mean QUALID score was 24.0 (SD 7.9) as assessed by family caregivers, and 25.3 (SD 8.6) as assessed by the nursing staff. Swallowing problems were the most prevalent dementia-related health problem (11.4%). Agitation was often reported by physicians (42.0%) and nursing staff (40.5%). Psychotropics were prescribed frequently (72.3%). A minority had written advance directives (5.4%) or documentation on treatment preferences by the former general practitioner (27.2%). Global care goals most often focused on comfort (73.9%). Proportions of do-not-treat orders were higher than do-treat orders for all interventions except for hospitalization and antibiotics.

**Conclusions:**

ACP must be initiated earlier, before nursing home admission. A palliative approach seems appropriate even though residents are relatively young and experience few dementia-related health problems.

## INTRODUCTION

Dementia-specific palliative care strategies are mainly based on research on persons with late-onset dementia (LOD) [1, 2]. It is unclear whether these strategies should also apply to people with young-onset dementia (YOD) [3], who develop symptoms before the age of 65 [4]. However, those with YOD could benefit from palliative care as it is aimed at improving the quality of life (QOL) of those facing problems associated with life-threatening illnesses [5].

Good palliative care for people with dementia comprises multiple domains according to the white paper by the European Association for Palliative Care [2]. The baseline is a palliative care approach that refers to all treatment and care, including adequate treatment of behavioral and psychological symptoms of dementia, comorbid diseases, and health problems. Palliative care also entails support for family members as may be needed in their role as proxy decision-makers and in dealing with the caregiving situation. Advance care planning (ACP) is one of the key components of palliative care in dementia. A recent umbrella review showed that ACP is associated with improved end-of-life outcomes [6]. ACP involves involves reflection and dialogue about preferences for future care between the patient and the healthcare team, but also with family caregivers who may continue the dialogue with the healthcare team if the patient can no longer be involved. It may include the completion of advance directives and discussion of treatment orders to anticipate future scenarios [7]. ACP continues to evolve and undergoes reconceptualization as a holistic, ongoing process throughout the life course, encompassing both tailored in-the-moment decisions and advance choices at every life stage [8].

It can be expected that the palliative care needs of people with YOD differ from people with LOD due to distinct characteristics of YOD including fewer somatic comorbidities [9], more neuropsychiatric symptoms [10], higher disease awareness [11], and larger caregiver burden [12]. However, palliative care needs remain underinvestigated despite a growing number of studies on people with YOD. The German EPYLOGE-study is the only study on this topic so far [13]. Its cohort consisted of people with advanced YOD and LOD living at home or in long-term care (LTC) facilities. Those younger than 65 years experienced symptoms that were significantly more distressing than older patients. Patients with YOD had fewer somatic comorbidities and were less frequently admitted to a hospital in the last three months of life [14]. There are no known reports on palliative care needs and provision in LTC settings that are specialized in the care of residents with YOD.

YOD special care units are available across the Netherlands [15]. The Young-Onset Dementia Knowledge Center, founded in 2013, supports healthcare organizations in providing age-appropriate and high-quality care for patients with YOD according to a National Care Program [16]. Therefore, the Dutch setting offers the opportunity to study palliative care practices in the unique context of infrastructure established for specialized nursing home care for young people living with dementia. The aim of this study is to describe health problems, symptoms, symptom treatment, and ACP, focusing on medical treatments, in a sample of residents with YOD living in special care units.

## METHODS

This paper reports baseline data of the observational Care4Youngdem-study (WHO International Clinical Trials Registry Platform (ICTRP) ID NTR5989, registration date 2016-09-08) which aims to map the practice of palliative care in YOD from three perspectives: physician or advanced nurse practitioner, family caregiver, and nursing staff. Questionnaires were administered after inclusion (baseline) and after death. Data collection started in 2016 and ended in 2022.

### Context

After admission to a LTC facility in the Netherlands, physicians or advanced nurse practitioners, employed by the LTC organization, provide medical care. Certified elderly care physicians who have specialized as primary care experts in geriatric medicine serve as attending physicians [17]. Daily nursing care is delivered by nurse assistants with three years of vocational training (European Qualification Framework (EQF) 3), nurse aides (EQF 2), and registered nurses (EQF 4–6) [18].

### Recruitment

Patient enrolment and collection of baseline data took place from 2016 to 2020. We approached 34 LTC organizations and recruited 16 (response 47%) with a total of 18 YOD special care units. All participating LTC organizations were affiliated with the Young-Onset Dementia Knowledge Center [16].

Members of the nursing home staff approached family caregivers and provided them with an information letter. All residents, regardless of their length of stay, were eligible for inclusion if 1) there was a physician diagnosis of dementia with first symptom onset before the age of 65 years; 2) family caregivers provided informed consent to participate and to transfer data about the resident to the researchers.

### Data collection

After receiving informed consent, the resident’s physician or advanced nurse practitioner, family caregiver, and nurse assistants or registered nurses were asked to complete the questionnaires (Table 1). Validated and translated assessment instruments were available mostly through research on palliative care in LOD [19].

**Table 1:** Overview of instruments and measures used including type of respondent.

Topic	Measurement instrument or question	Range or options	Respondent
Demographics			Physician
Relationship with the resident Date of diagnosis			Family caregiver Physician
Date of diagnosis
Dementia type		Alzheimer’s disease Frontotemporal dementia Vascular dementia Lewy Body Dementia or Parkinson dementia Alcohol-related dementia Mixed dementia of Alzheimer’s disease and vascular dementia Other dementia^a^ ^a^Other dementia was recoded if possible	Physician
Dementia severity	Global Deterioration Scale [20]	7 categories with elaborate verbal descriptions scored 1 - 7	Physician
Quality of life	The quality of life in late-stage dementia scale	1–5 per item with item-specific response options 11–55 total score with lower scores representing better quality of life	Family caregiver and nursing staff
Weight loss	Resident Assessment Instrument-Minimum Data Set 2.0 [56]	Present (body weight reduction 5% over last month or 10% over last six months) Absent	Physician
Nutritional status		Very cachectic^b^ Cachectic Normal Obese Very obese^c^ ^b^Very cachectic was recoded into cachectic ^c^Very obese was recoded into obese	Physician
Hydration status		Dehydrated Mildly dehydrated Normal	Physician
Swallowing problems		Present or absent	Nursing staff
Life expectancy	Surprise question: “Would I be surprised if the patient died in the next year?” [23]	Yes or no	Physician
Symptoms (pain and agitation)	Frequency in the last week	Yes, no and do not know Yes (2-3 days, 4-5 days, and 6-7 days) or no (<1 day or never)	Physician Nursing staff
Symptoms (various)	End-of-Life in Dementia Scale - Symptom Management [25]	Frequency categories scored 0 – 5 per item 0–45 total score	Family caregiver
Symptom treatment	Anatomical Therapeutic Chemical classification system [21]		Physician
Written advance directives		Present, absent or unknown	Physician
Documentation of the former general practitioner on treatment preferences Medical decision-making capacity of resident		Present, absent or unknown Yes Partly No	Physician Physician
Prioritized global care goal		1. Palliative, i.e., aimed at well-being and quality of life, irrespective of shortening or prolonging of life^d^ 2. Symptomatic, i.e., aimed at well-being and quality of life, additional prolonging of life undesirable^d^ 3. Maintaining or improving function 4. Life prolongation 5. Not yet determined 6. Other ^d^Palliative and symptomatic together constitute a comfort care goal	Physician
Treatment order	Resuscitation Hospitalization Admission to an intensive care unit Tube feeding Intravenous therapy Hypodermoclysis Antibiotics	Do-treat Do-not-treat Discussed but no order Not discussed and no order	Physician

The questionnaire included items on demographics, date of admission, type and severity of dementia, and date of diagnosis. The type of dementia was asked as stated in the medical record. The type was categorized into Alzheimer’s disease, vascular dementia, frontotemporal dementia, mixed dementia of Alzheimer’s disease dementia and vascular dementia, alcohol-related dementia, dementia by multiple causes, other causes of dementia, and dementia not otherwise specified. Dementia severity was assessed with the Global Deterioration Scale (GDS) [20]. This scale describes seven different stages of dementia and has been validated against behavioral, neuroanatomic, and neuropsychological measures. Medication overviews were provided to determine pain medication and psychotropics including pro re nata prescriptions. These were classified according to the Anatomical Therapeutic Chemical (ATC) classification [21].

Pre-structured items from the Resident Assessment Instrument-Minimum Data Set 2.0 [22] on nutritional status and hydration status were completed. Weight loss was defined as 5% over the last month or 10% over the last six months. Swallowing problems were assessed as either being present or absent. Physicians were asked the surprise question (“Would you be surprised if this patient died within the next 12 months?”) which is a frequently used screening tool to create awareness around identifying people nearing the end of life within various patient groups [23, 24].

The frequency of pain and agitation over the last week was assessed by both the physician and nursing staff. Symptoms during the previous month were assessed by family caregivers with the Dutch version of the End-of-Life in Dementia Scale - Symptom Management (EOLD-SM) [25]. It assesses the frequency of nine items: pain, shortness of breath, depression, fear, anxiety, agitation, calm, skin breakdown, and resistance to care. Higher scores (range 0 to 45) indicate better symptom control. The scale showed acceptable Cronbach’s *α* coefficients and approximately normal distributions in a Dutch nursing home cohort [26].

The Quality of Life in Late-Stage Dementia (QUALID) [27] was used as a proxy rating of QOL by the family caregiver and nurse (assistant). It comprises 11 items that assess observed well-being through the intensity of a resident’s behavior. The scale was initially developed for nurses. However, it can be administered by family caregivers who are familiar with the resident’s behavior [28]. Lower scores (range 11 to 55) indicate better QOL. The validated Dutch version of the QUALID showed good test-retest reliability and moderate interrater reliability [29].

Questions related to ACP included the presence of written advance directives, the existence of documents drawn up before admission by the former general practitioner (GP) on (future) treatment preferences of the person with YOD, the medical decision-making making capacity of the resident as assessed by the physician, and questions regarding treatment orders anticipating future scenarios. Care goals and treatment orders could be agreed upon by physicians and residents or family caregivers depending on the medical decision-making capacity of a resident. The prioritized care goal was presented with five possible pre-structured options and a sixth open-ended option. Treatment orders referred to resuscitation, hospitalization, admission to an intensive care unit, tube feeding, intravenous therapy, hypodermoclysis, and antibiotics. Most of these treatments are delineated within a Dutch primary care physicians guideline on ACP and also part of ACP-discussions in daily nursing home practice [30, 31]. Response options included do-treat, do-not-treat, discussed but no order, and not discussed and no order.

Some previous studies in the field of palliative care either exclusively enrolled individuals with advanced dementia or conducted analyses in a subgroup of those with advanced dementia [32, 33]. Therefore, we performed an additional analysis which included the QUALID scale and all characteristics related to palliative care and ACP of residents with advanced dementia, which was defined as a GDS score of 7.

### Statistical analysis

The statistical software SPSS version 25.0 was used to conduct statistical analysis (SPSS Inc, 2017, IBM, USA). Continuous variables were presented as mean with standard deviation and range. Categorical variables were represented as frequencies and corresponding percentages. In cases of no more than one-third of missing QUALID or EOLD-SM items, the total score was generated by imputing the mean patient item score.

### Ethical considerations

The study protocol (number 2016-2505) was reviewed and declared exempt from the Medical Research Involving Human Subjects Act (WMO) by the designated Medical Research Ethics Committee (Commissie Mensgebonden Onderzoek Regio Arnhem/Nijmegen) on 9 June 2016. The research project was performed in accordance with the principles of the Declaration of Helsinki and the Dutch Medical Treatment Contracts Act.

## RESULTS

A total of 215 family caregivers agreed to participate and collect data about the resident (Supplementary Figure 1). We report on 185 residents (86.0%) for which all three respondents (physicians, family caregivers, and nursing staff) completed the questionnaire.

### Patient and dementia-related characteristics

The mean age of residents with YOD was 63.9 years, ranging from 45 to 76 years (Table 2). Women comprised 50.3% of the residents. Most of the family caregivers were partners (55.7%). The average length of stay in long-term care was 2.3 years with a range from 10 days to 13 years.

**Table 2:** Patient and dementia-related characteristics of residents (N = 185)

	Number	%
**Age, y**
Mean (SD)	63.9 (5.8)
Range	45–76
**Sex**
Female	93	50.3
Male	92	49.7
**Family caregiver relationship**
Partner	103	55.7
Sibling	35	18.9
Child	29	15.7
Parent	2	1.1
Professional legal guardian	2	1.1
Other	14	7.6
**Length of stay in long-term care facility**
Mean (SD)	2.3 (2.4) y
Range	10 days-13.0 y
**Type, number (%)**
Alzheimer’s disease dementia	78	42.2
Frontotemporal dementia	37	20.0
Vascular dementia	13	7.0
Lewy Body Dementia or Parkinson dementia	10	5.4
Mixed dementia of Alzheimer’s and vascular dementia	8	4.3
Alcohol-related dementia	7	3.8
Dementia due to multiple causes^a^	7	3.8
Other causes of dementia^b^	15	8.1
Dementia not otherwise specified	10	5.4
**Time since diagnosis^**c**^**
Mean (SD)	4.8 (3.1) y
Range	5 mo–22.7 y
**GDS^**d**^**
≤4, No to moderate cognitive decline	21	11.4
5, Moderately severe cognitive decline	47	25.5
6, Severe cognitive decline	67	36.4
7, Very severe cognitive decline	49	26.6
**QUALID^**e**^**
**Score assessed by family caregivers^**f**^**
Mean (SD)	24.0 (7.9)
Range	11–47
**Score assessed by nursing staff**
Mean (SD)	25.3 (8.6)
Range	11–48

^a^Combination of vascular and frontotemporal dementia (N = 3), Alzheimer’s disease dementia and frontotemporal dementia (N = 2), Alzheimer’s disease dementia and Lewy body dementia (N = 1), and vascular dementia and alcohol-related dementia (N = 1). ^b^Posterior cortical atrophy (N = 2) and various other types of dementia (all N = 1). ^c^N = 179. ^d^Global deterioration Scale, N = 184. ^e^Quality of life in late-stage dementia scale; lower scores indicate better quality of life. ^f^N = 183; N = 2 imputed values.

Alzheimer’s disease dementia had the highest prevalence (42.2%) followed by frontotemporal dementia (20.0%) and vascular dementia (7.0%). The majority of residents experienced severe or very severe cognitive decline (36.4% and 26.6%, respectively). However, moderate (8.7%) and moderately severe cognitive decline (25.5%) were not rare. Family caregivers rated QOL slightly higher than nursing staff as indicated by lower QUALID scores on average: 24.0 (SD 7.9) versus 25.3 (SD 8.6). In case of advanced dementia, mean QUALID scores were 24.8 (SD 8.7) and 28.6 (SD 9.2), respectively.

### Palliative care-related characteristics

Only 8.1% of residents experienced weight loss (Table 3). Cachexia was present in 8.7% of residents. The prevalence of obesity was 15.8%. Dehydration was uncommon (1.1%). Difficulties with swallowing occurred in 11.4%. In over half (56.5%) of cases the physician would be surprised if the resident were to die within a year.

**Table 3:** Palliative care-related characteristics of residents

	All residents (N = 185)		Residents with advanced dementia (N = 49)	
Number	%	Number	%
**Dementia-related health problems**
**Weight loss**
Present	15	8.1	10	20.4
Absent	160	86.5	46	73.5
Unknown	10	5.4	3	6.1
**Nutritional status^a^**
Cachectic	16	8.7	11	22.4
Normal	139	75.5	33	67.3
Obese	29	15.8	5	10.2
**Hydration status^a^**
Dehydrated	2	1.1	2	4.1
Mildly dehydrated	5	2.7	4	8.2
Normal	177	96.2	43	87.8
**Swallowing problems**
Present	21	11.4	9	18.4
Absent	164	88.6	40	81.6
**Surprise question^a,*b*^**
Yes	104	56.5	13	26.5
No	80	43.5	36	73.5
**Symptoms in the previous week**
**Pain**
Yes, according to physician^c^	33	18.6	7	15.6
Yes, according to nurse	56	30.2	13	26.5
**Agitation**
Yes, according to physician^d^	73	42.0	26	53.1
Yes, according to nurse	75	40.5	24	49.0
**EOLD - Symptom Management^e^**
Mean (SD)	31.9 (8.5)		31.5 (9.0)
Range	10–45		11–45
**Pain medication (total of M01A, N02A, and N02BE01)^f^**	**51**	**29.5**	**16**	**34.8**
NSAIDs (M01A)	5	2.7	1	2.2
Opioids (N02A)	13	7.0	4	8.7
Paracetamol (N02BE01)	42	24.3	13	28.3
**Psychoactive medication (total of N03A, N05, N06A, and N06D) ^f,*g*,*h*^**	**125**	**72.3**	**34**	**73.9**
Antipsychotics (N05A)	72	41.6	18	39.1
Anxiolytics (N05B)	33	19.1	10	21.7
Antidepressants (N06A)	69	39.9	19	41.3

^a^N = 184. ^b^Answer to the question: “Would I be surprised if the patient died within one year?”. ^c^N = 177 and 45. ^d^N = 174 and 49. ^e^End-of-Life in Dementia scales, N = 182; detailed information can be found in Supplementary Table 1. ^f^N = 173 and 46 (12 missing medication overviews). ^g^N03A comprises antiepileptics. ^h^N06D comprises antidementia drugs.

Pain was frequently reported by the nursing staff and physician (30.2% and 18.6%, respectively). Agitation was observed in almost half of cases (40.2% and 42.0%, respectively). The mean EOLD-SM total score as assessed by family caregivers was 31.9 (SD 8.5). The family caregivers had observed agitation (34.6%) and pain (25.2%) at least once a week over the previous month (Supplementary Table 1). Skin breakdown (4.8) and shortness of breath (4.5) had the highest mean values of the EOLD-SM of all items indicating that in most cases these symptoms had not occurred in the last month (91.2% and 82.0%, respectively). Anxiety had the highest mean score (2.7), 41.5% of residents experienced this symptom at least once a week. Pain medication was administered to 29.5% of residents. Almost three-quarters (72.3%) of the residents were prescribed at least one psychotropic drug, mostly antipsychotics (41.6%) and antidepressants (39.9%). Residents with advanced dementia experienced more often agitation and a higher symptom burden as shown by lower EOLD-SM scores. Malnutrition, cachexia, and dehydration were more prevalent.

### ACP-related characteristics

Written advance directives were present in 5.4% of residents (Table 4). Documentation of the former GP specifically on treatment preferences was available in 27.2% of cases. In around a third of cases (34.2%), the physician deemed the resident at least partly able to make decisions regarding medical treatments.

**Table 4:** ACP-related characteristics of residents

	All residents (N = 184)		Residents with advanced dementia (N = 49)	
	Number	%	Number	%
**Written advance directive (living will)**
Present	10	5.4	3	6.1
Absent	160	87.0	44	89.8
Unknown	14	7.6	2	4.1
**Documentation of the former general practitioner on treatment preferences**
Present	50	27.2	14	28.6
Absent	119	64.7	28	57.1
Unknown	15	8.2	7	14.3
**Medical decision-making capacity of resident**
Yes	10	5.4	2	4.1
Partly	53	28.8	1	2.0
No	121	65.8	46	93.9
**Care goal**
Palliative^a,c^	114	62.0	34	69.4
Symptomatic^b,c^	22	12.0	9	18.4
Maintaining or improving function	26	14.1	2	4.1
Life prolongation	16	8.7	3	6.1
Other	4	2.2	1	2.0
Undecided	2	1.1	0	0.0

^a^Definition: aimed at well-being and quality of life, irrespective of shortening or prolonging of life. ^b^Definition: aimed at well-being and quality of life, additional prolonging of life undesirable. ^c^Palliative and symptomatic together constitute a comfort care goal.

**Fig. 1:**
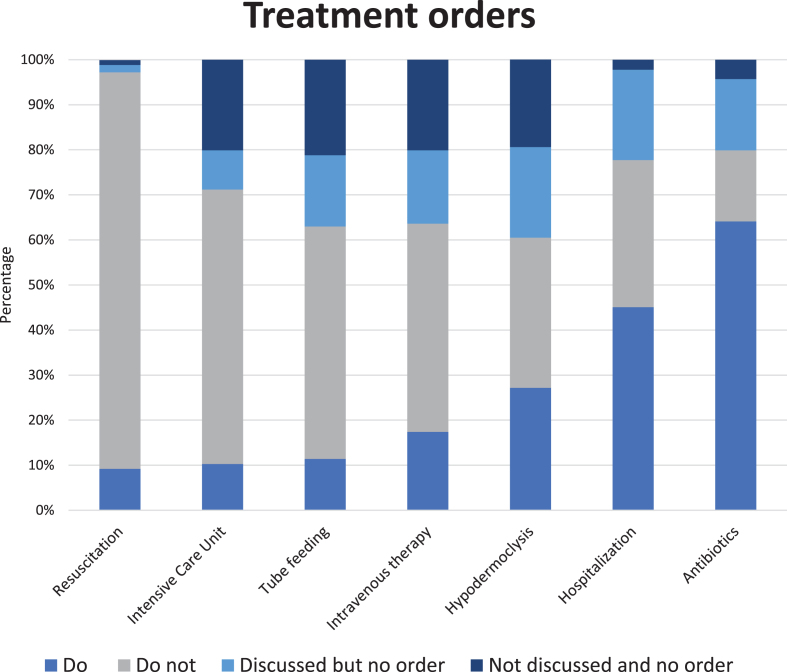
Percentages (N = 184) of specific advance treatment orders. Percentage ‘do-not-treat’ order per type of treatment in descending order: resuscitation 88.0%, intensive care unit 60.9%, tube feeding 51.6%, intravenous therapy 46.2%, hypodermoclysis 33.3%, hospitalization 32.6%, and use of antibiotics 15.8%; we refer to Supplementary Table 2 for percentages of all answer options, including from residents with advanced dementia, presented separately.

A global care goal was established for 98.9% of residents. For the majority of residents (73.9%), the prioritized care goal was comfort. Maintaining or improving function was the main goal for 14.1% and life prolongations for 8.7% of residents. Do-not-treat orders (Fig. 1 and Supplementary Table 2) were most common with regard to resuscitation (88.0%) and admission to an intensive care unit (60.9%). Lower percentages were found for tube feeding (51.6%), intravenous therapy (46.2%), hypodermoclysis (33.3%), hospital admission (32.4%), and use of antibiotics (15.8%). Resuscitation had the lowest percentage of ‘no order’ (2.7%) in which a decision had not yet been made and tube feeding advance orders were set least often (37.0%). The physician and family caregiver had almost always discussed resuscitation (98.9%), hospitalization (97.8%), and antibiotics (95.7%), in contrast to the other types of treatment. Residents with advanced dementia (Table 4 and Supplementary Table 2) more often had a comfort care goal (87.8%). The proportion of do-no-treat orders was higher for all types of treatment as compared the whole cohort.

## DISCUSSION

This is the first study on palliative care-related characteristics of a group of residents living in YOD special care units in the Netherlands from three perspectives. Dementia severity, dementia type, and quality of life varied. Health problems, of which swallowing problems were most frequently reported, were relatively uncommon. However, neuropsychiatric symptoms were reported often. Psychotropic drugs, antipsychotics in particular, were prescribed frequently. Advance directives and ACP agreements before admission were uncommon, yet for the large majority of residents, comfort care goals and do-not-treat orders were established afteradmission.

QOL can be considered one of the main objectives of palliative care. It has been studied in YOD with the use of different proxy ratings including the QUALID scale [13]. In our population, mean QUALID scores (24.0) as assessed by family caregivers were higher than in the EPYLOGE cohort (21.0), pointing to lower observed QOL in our sample. The higher QUALID scores are consistent with more frequent antipsychotic drug treatment and neuropsychiatric symptoms such as fear, agitation, and lack of calmness in our cohort as compared to EYPLOGE, in which these factors were found to be negatively associated with QOL. High neuropsychiatric symptom burden was observed by family caregivers as indicated by low mean values of EOLD-SM items referring to psychological and behavioral symptoms. Agitation was common according to physicians and nursing staff. High prevalence rates of neuropsychiatric symptoms in our study, despite the frequent use of psychotropic drugs, is in line with previous research on nursing home resident with YOD [34]. Psychosocial interventions must also be considered since positive effects have been shown in dementia care [35]. An international panel agreed that non-pharmacological interventions are often the most appropriate way of treating behavioral symptoms in YOD [36]. However, it is unclear which YOD-tailored interventions are effective.

One of the domains of palliative care is providing prognostic information [2]. Recognition of dementia-related health problems is relevant as these may affect prognosis [33, 37, 38]. It can guide the physician in initiating ACP discussions [39]. Previous studies have also indicated that persons with YOD and caregivers wish to have a better understanding of symptom progression and prognosis [40]. Malnutrition, indicated by cachexia or weight loss, occurred less frequently than in the general Dutch LTC population in which numbers have remained relatively stable over the years [41]. The prevalence rates of dehydration and swallowing problems were also lower than those found in older nursing home populations [42, 43]. Prevalence of swallowing problems was highest among dementia-related health problems, which may be relevant as swallowing difficulty was the most frequently observed symptom at the end of life in EPYLOGE [14]. The relatively low prevalence rates of dementia-related health problems could point to better physical fitness of residents with YOD compared to other nursing home residents. Despite low frequencies of these somatic health problems, in almost half of cases the physician would not be surprised if the person with YOD were to die within 12 months. Therefore, physicians may be taking other scenarios into account, such as cognitive deterioration or the possibility of acquiring pneumonia, when estimating life expectancy.

Advance directives, written by people with YOD themselves, were not common in our study (5.4%). The lack of advance directives can be considered problematic as a strong association has been found between having a written advance directive and the quality of dying for nursing home residents with dementia [44]. This low number is also in contrast to the hypothesis that people with YOD have different attitudes compared to those with LOD toward their disease and prognosis, asking for autonomy in the decision-making process, and playing an active role in ACP [3, 40]. Advance directives were more common in studies conducted in Germany (70%) and the United States (17%) for both YOD and LOD indicating that cultural differences play a role [14, 45]. Cultural differences concerning ACP, partly because of different legal frameworks, have been described in qualitative YOD research [46]. American caregivers relied on legal professionals from nonmedical domains for guidance in correctly completing living wills whereas Belgian patients and caregivers turned to healthcare professionals, such as GPs.

No document of the former GP was available on treatment preferences in more than half of cases (64.7%). Relatedly, in Dutch primary care ACP conversations between GPs and people with dementia are rare [47]. There are fewer end-of-life and ACP discussions compared to cases of people with cancer or organ failure [48]. Important barriers found in a systematic review were [49]: uncertainty about the timing, lack of knowledge about dementia, difficulties assessing decisional capacities of people with dementia, and changing preferences.

Uncertainty about timing due to a better health status could also play a role in YOD [9, 39]. A gap of knowledge hindered ACP engagement in YOD according to qualitative research from Flanders as well [50]. In this study, doubt regarding cognitive ability was also identified as a reason not to initiate ACP. In our cohort, around two thirds of residents were assessed as being unable to make decisions regarding medical treatments. Another possible explanation for low prevalence of ACP conversations and documentation in the YOD population was the the unpredictability of the disease trajectory which can make couples feel unprepared [50, 51].

A Belgian interview study illustrated that patients with YOD and caregivers nevertheless recognize the potential benefits of ACP [52]. One frequently emerging motivation for engaging in ACP was that persons with YOD would be enabled to participate in decision-making. A second reason was found in the relief that planning in advance would bring to the caregivers. Our study focuses on future medical care as part of ACP. However, it was found that an overly medicalized approach might impede people’s engagement. Therefore, implementation as a holistic, flexible, and relational communication process is suggested [52].

In most cases family caregivers and physicians established a comfort care goal (73.9%). A lower percentage (64.7%) was found in the DEOLD study which included almost exclusively older nursing home residents with dementia [53]. The prevalence of do-not-resuscitate orders (88.0%) is in line with previous Dutch long-term care studies [54]. In our study, do-not-treat orders were more common than do-treat orders, except for hospitalization and antibiotics. In case of advanced dementia do-not-treat orders were even more common. The high numbers of comfort care goals and do-not-treat orders may seem counterintuitive given the relatively young age of the YOD population. Admission to a long-term care unit may be the appropriate time for caregivers to discuss ACP and to choose a palliative approach.

### Methodological considerations

One of the strengths of our study is the use of validated instruments that allow for comparison with other populations. Further, we report on the perspectives of physicians, family caregivers, and nursing staff, all of which are relevant to palliative care research. Different scores such as QUALID were found for different respondents, which could be due to differences in reference period or level of involvement with the resident.

Our findings may reflect characteristics specific to the Dutch context in which LTC organizations are stimulated to improve dedicated services for YOD, for example by adapting a multidisciplinary approach and improving team expertise through staff training [16]. Different practices, not tailored to YOD, could be common elsewhere perhaps related to different palliative care outcomes.

Physicians were asked to report the dementia type as stated in the medical record without detail on diagnostic work-up. Atypical combinations of dementia types were seen. Therefore, the diagnostic accurateness could be questioned. However, most residents probably had received adequate work-up to determine the type of dementia as was found in a Dutch study with a similar nursing cohort [15]. Moreover, the Dutch dementia guideline also recommends to refer younger patients to a memory clinic [55].

### Conclusion and implications

Our study describes a sample of people with YOD residing in YOD special care units. Dementia-related health problems were less common than in the general nursing home population. A palliative care approach should focus on neuropsychiatric symptoms; particularly fear, agitation, and resistiveness to care are relevant as these are more prevalent than physical symptoms. Advance directives and ACP agreements before admission to long-term care were low despite the necessity of timely discussion of care preferences between people with YOD, caregivers, and professionals. After admission, on the other hand, ACP practices were common in the form of care goals and treatment orders. Despite their relatively young age and the low number of dementia-related health problems, most YOD residents had comfort care goals and do-not-treat orders. Therefore, a palliative approach can be considered appropriate for most people with YOD. This realization could help GPs and other professionals in timely discussion of ACP. They should fulfill an active role in initiating ACP conversations to ensure that future palliative care is in accordance with the person’s wishes. Future studies may examine the impact of timely implementation of ACP on quality of care and life.

## Supplementary Material

Supplementary Material

## Data Availability

The data supporting the findings of this study are available upon reasonable request from the corresponding author.
