# Supplementary material for: Palliative Care in Nursing Home Residents with Young-Onset Dementia: Professional and Family Caregiver Perspectives
Source: J Alzheimers Dis. 2024 Jan 16;97(2):573–86. doi: 10.3233/JAD-230486 (PMC10836558; doi:10.3233/JAD-230486)
Supplement: Supplementary Material [file jad-97-jad230486-s001.pdf]

Supplementary Material

Palliative Care in Nursing Home Residents with Young-Onset Dementia: Professional and Family Caregiver Perspectives

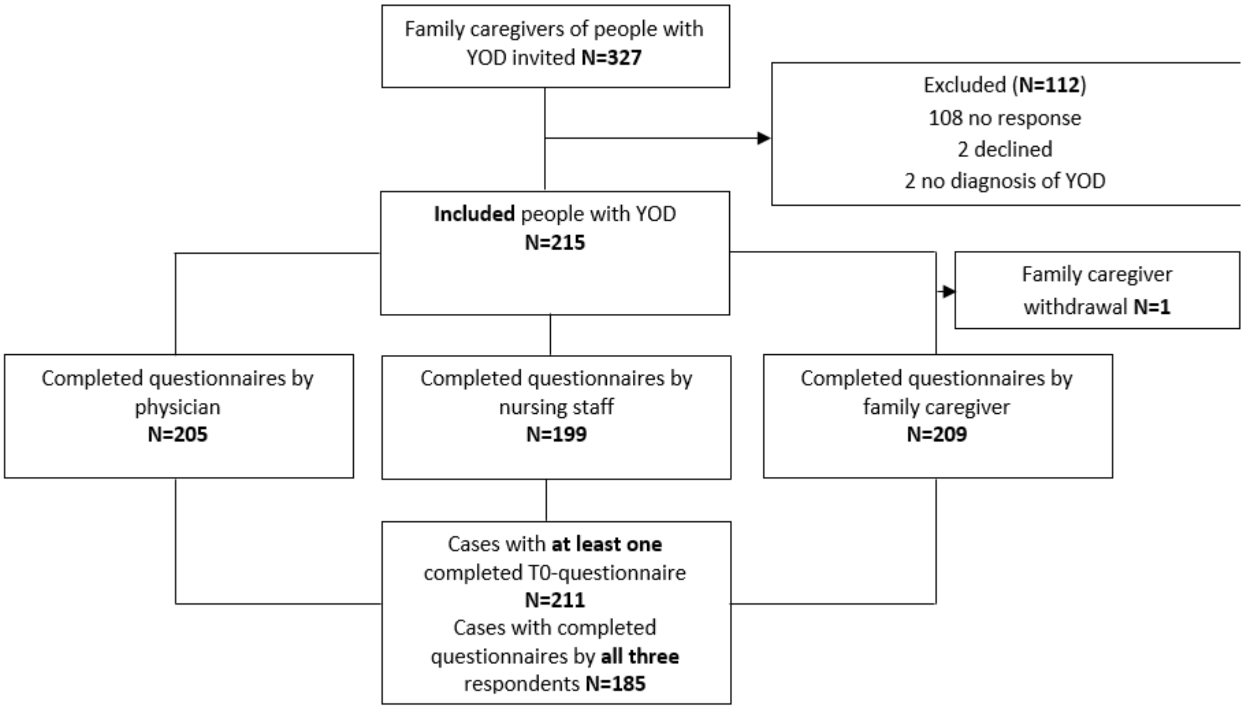

Supplementary Figure 1. Flowchart of inclusion and baseline assessments

Supplementary Table 1. End-of-Life in Dementia scales-Symptom Management (N=182)

**Score**<br><br>**Label**	**0**<br>**Every day**	**1**<br>**2 or 3 days a week**	**2**<br>**Once a week**	**3**<br>**Several days a month**	**4**<br>**Once a month**	**5**<br>**Never**	**Mean (SD)**
Pain<br>(% of 183)	6.6	12.6	6.0	16.9	24.0	33.9	3.3 (1.7)
Shortness of breath<br>(% of 183)	2.7	4.9	4.4	2.7	3.3	82.0	4.5 (1.2)
Skin breakdown<br>(% of 182)	1.1	2.7	0	2.2	2.7	91.2	4.8 (0.9)
Calm*<br>(% of 181)	53.6	5.0	1.7	26.0	1.1	12.7	3.9 (1.7)
Depression<br>(% of 182)	7.1	9.3	7.7	15.4	4.9	55.5	3.6 (1.8)
Fear<br>(% of 183)	10.9	8.2	13.7	12.6	13.7	41.0	3.2 (1.8)
Anxiety<br>(% of 183)	19.1	10.9	11.5	17.5	7.7	33.3	2.7 (2.0)
Agitation<br>(% of 182)	9.9	12.1	12.6	20.9	12.1	32.4	2.9 (1.8)
Resistiveness to care<br>(% of 181)	9.4	7.7	13.8	22.1	11.0	35.9	3.0 (1.9)

Reference: Volicer L, Hurley AC, Blasi ZV (2001) Scales for evaluation of End-of-Life Care in Dementia. *Alzheimer Dis Assoc Disord* **15**, 194-200.

* Calm is reverse coded for calculation of the score

Table S2 - Treatment orders results (N = 184)

	All residents		Residents with severe dementia (N = 49)	
**Treatment orders**	**Frequency**	**%**	**Frequency**	**%**
**Resuscitation**				
Do	17	9.2	1	2.0
Do not	162	88.0	48	98.0
Discussed but no order	3	1.6	0	0
Not discussed and no order	2	1.1	0	0
**Intensive Care Unit**				
Do	19	10.3	4	8.2
Do not	112	60.9	38	77.6
Discussed but no order	16	8.7	0	0
Not discussed and no order	37	20.1	7	14.3
**Tube feeding**				
Do	21	11.4	1	2.0
Do not	95	51.6	30	61.2
Discussed but no order	29	15.8	10	20.4
Not discussed and no order	39	21.2	8	16.3
**Intravenous therapy**				
Do	32	17.4	1	2.0
Do not	85	46.2	33	67.3
Discussed but no order	30	16.3	9	18.4
Not discussed and no order	37	20.1	6	12.2
**Hypodermoclysis**				
Do	50	27.2	8	16.3
Do not	61	33.2	23	46.9
Discussed but no order	37	20.1	11	22.4
Not discussed and no order	36	19.6	7	14.3
**Hospitalization**				
Do	83	45.1	11	22.4
Do not	60	32.6	28	57.1
Discussed but no order	37	20.1	10	20.4
Not discussed and no order	4	2.2	0	0
**Antibiotics**				
Do	118	64.1	28	57.1
Do not	29	15.8	11	22.4
Discussed but no order	29	15.8	9	18.4
Not discussed and no order	8	4.3	1	2.0
